# Predilection for developing a hematogenous orthopaedic implant-associated infection in older versus younger mice

**DOI:** 10.1186/s13018-021-02594-0

**Published:** 2021-09-14

**Authors:** John M. Thompson, Alyssa G. Ashbaugh, Yu Wang, Robert J. Miller, Julie E. Pickett, Daniel L. J. Thorek, Robert S. Sterling, Lloyd S. Miller

**Affiliations:** 1 Department of Orthopaedic Surgery, Johns Hopkins University School of Medicine, Baltimore, MD 21287 USA; 2 School of Medicine, University of California Irvine, Irvine, CA 92697 USA; 3 Department of Dermatology, Johns Hopkins University School of Medicine, Baltimore, MD 21287 USA; 4 Eshelman School of Pharmacy, University of North Carolina at Chapel Hill, Chapel Hill, NC 27599 USA; 5 Division of Radiological Chemistry and Imaging Laboratory, Washington University School of Medicine, St. Louis, MO 63110 USA; 6 Immunology, Janssen Research and Development, 1400 McKean Road, Spring House, PA 19477 USA

**Keywords:** Hematogenous, Orthopaedic, Implant, Infection, *Staphylococcus aureus*

## Abstract

**Background:**

The pathogenesis of hematogenous orthopaedic implant-associated infections (HOIAI) remains largely unknown, with little understanding of the influence of the physis on bacterial seeding. Since the growth velocity in the physis of long bones decreases during aging, we sought to evaluate the role of the physis on influencing the development of *Staphylococcus aureus* HOIAI in a mouse model comparing younger versus older mice.

**Methods:**

In a mouse model of HOIAI, a sterile Kirschner wire was inserted retrograde into the distal femur of younger (5–8-week-old) and older (14–21-week-old) mice. After a 3-week convalescent period, a bioluminescent *Staphylococcus aureus* strain was inoculated intravenously. Bacterial dissemination to operative and non-operative legs was monitored longitudinally *in vivo* for 4 weeks, followed by *ex vivo* bacterial enumeration and X-ray analysis.

**Results:**

*In vivo* bioluminescence imaging and *ex vivo* CFU enumeration of the bone/joint tissue demonstrated that older mice had a strong predilection for developing a hematogenous infection in the operative legs but not the non-operative legs. In contrast, this predilection was less apparent in younger mice as the infection occurred at a similar rate in both the operative and non-operative legs. X-ray imaging revealed that the operative legs of younger mice had decreased femoral length, likely due to the surgical and/or infectious insult to the more active physis, which was not observed in older mice. Both age groups demonstrated substantial reactive bone changes in the operative leg due to infection.

**Conclusions:**

The presence of an implant was an important determinant for developing a hematogenous orthopaedic infection in older but not younger mice, whereas younger mice had a similar predilection for developing periarticular infection whether or not an implant was present. On a clinical scale, diagnosing HOIAI may be difficult particularly in at-risk patients with limited examination or other data points. Understanding the influence of age on developing HOIAI may guide clinical surveillance and decision-making in at-risk patients.

## Introduction

Orthopaedic implant-associated infections (OIAI) arising from a hematogenous source are challenging to diagnose and treat, with little known about its pathogenesis. Hematogenous orthopaedic infection can occur both in an articular implant, such as a prosthetic joint infection (PJI), and/or in a non-articular orthopaedic implant; thus*,* OIAI acronym was chosen as a more general and accurate description. Hematogenous OIAI (HOIAI) is caused by the seeding of an infecting organism from a distant anatomical site through the blood stream to a surgically placed orthopaedic implant [1]. HOIAI are clinically significant as they are responsible for 20% of all OIAI, including 20–35% of prosthetic joint infections [2, 3]. These infections are most commonly caused by *staphylococcal* and *streptococcal* species [2, 4], although other infecting organisms that cause bacteremia and sepsis can also be involved. Typically, the original anatomic site of infection that result in the development of an ensuing HOIAI includes the skin and soft tissue, oral cavity, and urinary and gastrointestinal tracts [2, 5]. Clinical suspicion for HOIAI should be raised when OIAI arises greater than 1 month after the index procedure with a symptom-free intervening period and onset of acute pain [2, 5]. Since the number of orthopaedic surgical procedures are increasing, there is an unmet need to better understand the clinical features that predispose to the development of HOIAI, as well as the pathogenesis, diagnosis, and treatment [6–8].

The metaphyseal portion of long bones is typically the primary site for the seeding of hematogenous musculoskeletal infections [9–12]. The physis, which is located within the metaphyseal portion, is the center at which endochondral ossification occurs to promote long bone growth in the developing skeleton [13]. This region of the bone is highly vascular due to the high cellular metabolic activity required for new bone formation [14–18]. Although initially it was thought that osteomyelitis developed in long bones due to sluggish blood flow in the capillary loops [19, 20], it is now clearer that acute hematogenous osteomyelitis typically arises in the metaphysis of long bones from terminal metaphyseal vessels that are open ended and permit bacterial entry [10, 12, 21]. Blood flow rates are also significantly higher in the metaphyseal compared to diaphyseal or epiphyseal regions [22, 23]. As the long bones decrease their growth rate during transition into adulthood, the metabolic requirements decrease, and there are concomitant changes in vascular patterns with reduced blood flow in the metaphyseal region [22, 23].

Clinically, hematogenous osteomyelitis to the long bones is a substantial concern in pediatric patients [9–12] but occurs relatively infrequently in adult patients, as adults more commonly suffer spine infections in the setting of invasive procedures, diabetes, hemodialysis or immunodeficiency [24, 25]. Conversely, orthopaedic implant-associated infection is a feared complication more often in adult rather than pediatric patients. It is unclear whether similar differences associated with age influence the predilection of developing HOIAI. There have been several prior preclinical models of HOIAI in mice [26], rats [27], and rabbits [28–30], but none have specifically evaluated the impact of age in the development of HOIAI. Therefore, we set out to determine whether age was an important factor in the predilection for developing a hematogenous infection in younger versus older mice in an experimental model of HOIAI that employed noninvasive *in vivo* bioluminescence imaging (BLI) as well as *ex vivo* colony forming unit (CFU) enumeration and X-ray analysis. Based on clinical observations in adult and pediatric orthopaedic patients, we hypothesized that older mice would have a greater predilection for HOIAI, whereas younger mice would show a higher tendency for hematogenous osteomyelitis.

## Materials and methods

All work was performed at the Johns Hopkins University School of Medicine (Baltimore, MD 21287, USA).

### Bacteria

The stably bioluminescent methicillin-resistant *Staphylococcus aureus* (MRSA) strain SAP231, which was previously generated by genetic modification from the clinical USA300 community-acquired MRSA isolate NRS384 as previously described [31], was used in all experiments. This strain contains a *lux* operon that is stably integrated into the bacterial chromosome that is maintained in all progeny. Only live and actively metabolizing bacteria emit a bioluminescent signal. Bacteria were streaked onto tryptic soy agar (TSA) plates that were cultured overnight (16 h) in a bacterial incubator at 37 °C. Bacterial CFU were selected and cultured overnight in tryptic soy broth (TSB) at 37 °C with shaking (240 rpm). A 2-h subculture of a 1:50 dilution of the overnight culture was performed, and the bacteria were pelleted, washed, and reconstituted in sterile PBS.

### Mouse model of HOIAI

All mouse studies were approved by the Animal Care and Use Committee of this institution according to the guidelines and regulations described in the Guide for the Care and Use of Laboratory Animals (National Academies Press, 2011). All mice were maintained and housed under specific pathogen-free conditions at our animal facility accredited by the American Association for the Accreditation of Laboratory Animal Care (AAALAC) at this institution. Cages were stored in racks with automated water system, and mouse kibble was readily available. Older (14–21-week-old) and younger (5–8-week-old) male C57BL/6 mice (Jackson Laboratories, Bar Harbor, ME, USA) were housed for a 2-week acclimatization period grouped per age in the same facility under identical conditions after directly being purchased from the vendor. For this study, a previous mouse model of a *S. aureus* HOIAI model was employed [26], with group sizes of approximately 25 animals selected based on prior experience with the hematogenous infection model. Mice were anesthetized (2% isoflurane) and prepped for sterile surgery on the right legs by shaving and sterilizing the skin with 70% ethanol and betadine. An orthopaedic-grade titanium Kirschner-wire (K-wire) (Modern Grinding, WI, USA) was placed retrograde into the distal right femoral canal via a medial parapatellar approach. After a convalescent period of 3 weeks (at which point the postoperative inflammation returned to baseline [32]), an inoculum of SAP231 (1 × 10^7^ CFU in 100 μL of PBS) was injected intravenously via the retro-orbital vein in fully anesthetized mice as previously described [26]. Mice were then monitored by using *in vivo* bioluminescent imaging and by measuring weekly weights for 4 weeks before they were euthanized for X-ray imaging and *ex vivo* CFU enumeration. Body weights served as a proxy for systemic infection to monitor the overall health of the animals. In all experimental groups, this inoculum was 10–15% of the lethal dose (LD10-15), and those 10–15% of mice that succumbed from sepsis were excluded from all subsequent analyses. Mice were assessed daily during the acute post-infection period and were checked every few days before and after the acute post-infection period once body weights rebounded. Regarding the LD10-15, mice were prematurely euthanized and withdrawn from the study if they were moribund and/or had signs and symptoms of distress (e.g., hunched posture, inability to remain upright, lethargy, dehydration, respiratory distress, impaired mobility*,* and limited ability to obtain food or water). Mice also would have been prematurely euthanized if they had complications from the retro-orbital injection (e.g., proptosis, blepharitis, uveitis, and/or corneal ulceration), but none had these complications.

### *In vivo* BLI

*In vivo* BLI was performed on the mice on days 0, 3, 7, 14, 21, and 28 using the IVIS Lumina III (PerkinElmer, Hopkinton, MA, USA) with BLI exposure settings of large binning, f/stop of 1, and a 5-min exposure. *In vivo* BLI signals centered on the operative (op) right legs and contralateral non-operative (non-op) left legs of the mice were acquired using a 0.3 × 0.3-cm region of interest. Data were reported as mean maximum flux (photons per second per centimeter squared per steradian [photons/s/cm^2^/sr]) ± standard error of the mean (s.e.m.). The limit of detection of BLI is 2.5 × 10^3^ photons/s/cm^2^/sr [33], which we previously determined to be between 1 × 10^2^ and 1 × 10^3^ CFU [34].

### *Ex vivo* CFU enumeration

The experiment was arbitrarily ended on day 28 after the intravenous inoculation. All mice were euthanized, and the bone/joint tissues from the op and contralateral non-op legs as well as the implants were harvested. The bone/joint tissues from each of the op and non-op legs were homogenized (PRO200 Series; PRO Scientific, Oxford, CT, USA), and the tissue homogenates were then serially diluted and cultured overnight on plates at 37 °C. The implants from each of the op legs were sonicated in a 0.3% Tween (Sigma-Aldrich, St. Louis, MO, USA) solution for 10 min, vortexed for 2 min, and then serially diluted and cultured overnight on plates at 37 °C. To further distinguish between the presence and absence of an infection, the original tissue homogenates and implant sonicates were incubated in TSB for 48 h at 37 °C with shaking (240 rpm), followed by overnight plating of the broth at 37 °C. Growth in broth and plates were confirmed with BLI of the plates to evaluate for any contamination.

### X-ray imaging

On day 28, mice were euthanized and anteroposterior (AP) X-ray imaging of the op and contralateral non-op legs was performed using a Faxitron MX-20 (Faxitron Bioptics, Tucson, AZ, USA). The femoral length and width were measured using Image J image analysis software (https://imagej.nih.gov/ij/; National Institutes of Health). The femoral length was obtained by measuring the distance along the medullary line from the tip of the trochlea distally to its intersection with perpendicular line that bisects the third trochanter proximally. The femoral width was measured by identifying a line centered within the medullary canal and finding the maximum width proximal to the fabella perpendicular to this medullary line.

### Statistics

Group sizes of 20–25 mice were chosen based on our prior experience with the HOIAI mouse model to ensure statistical significant differences in the endpoints assessed [26]. Data for longitudinal *in vivo* BLI were compared using a 2-way ANOVA. Data for single comparisons (body weight and *ex vivo* CFU) were compared using a Mann-Whitney *U* test (2-tailed). The presence/absence of infection was calculated using a 2-tailed Fischer’s exact test. All statistics were calculated with Prism software (Graphpad; La Jolla, CA, USA). *P* < 0.05 was considered statistically significant.

## Results

### *In vivo* bacterial burden between younger and older mice

**Fig. 1:**
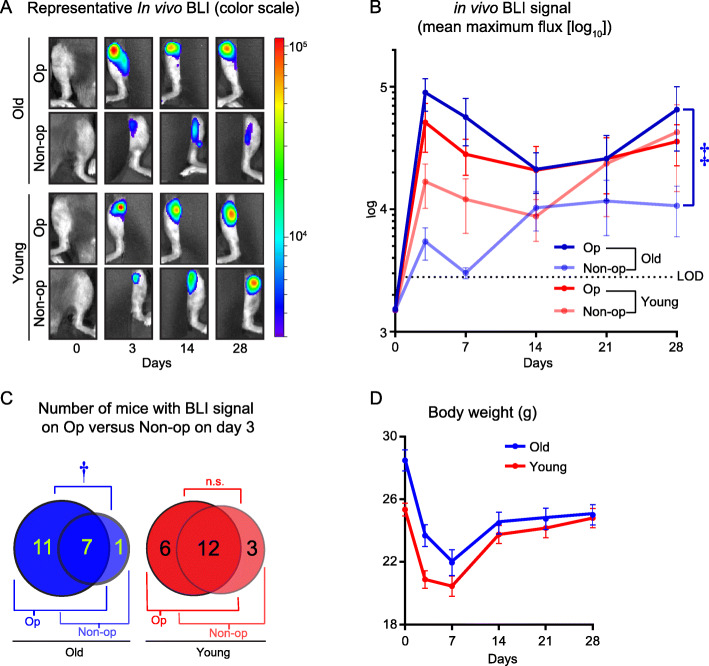
*In vivo* noninvasive monitoring of bacterial dissemination and burden in older and younger mice HOIAI. Three weeks after sterile surgical placement of an orthopaedic K-wire in the right distal femurs of older and younger mice (*n* = 19–21 mice/group), a bioluminescent MRSA strain was inoculated intravenously and *in vivo* BLI signals for the operative (op) and contralateral non-operative (non-op) legs were measured along with body weights for 28 days. **a** Representative *in vivo* BLI imaging (log scale). **b** Mean maximum flux (photons/s/cm^2^/sr) ± s.e.m. LOD = level of detection (3.3 × 10^3^ photons/s/cm^2^/sr). **c** Euler diagram depicting the number of mice with and/or without detectable *in vivo* BLI signals above the LOD in the op, non-op or both op and non-op legs. **d** Mean body weight (grams) ± s.e.m. *P < 0.05, †*P* < 0.01, and ‡*P* < 0.001, between op versus non-op for older and younger groups of mice, as calculated by 2-way ANOVA across all time points (**b**), 2-tailed Fischer’s exact test (**c**) or 2-tailed Mann-Whitney *U* test (**d**). *n.s.* not significant

Older mice (14–21 weeks old) (*n* = 24) and younger mice (5–8 weeks old) (*n* = 25 mice) had sterile surgical placement of an orthopaedic titanium K-wire implant retrograde in their right distal femoral canals via a medial parapatellar approach. Following a 3-week convalescent period at which time the acute inflammation from the sterile surgery resolved [32], a bioluminescent MRSA strain (SAP231) was injected intravenously in the retro-orbital vein to induce bacteremia, as previously described [26]. In the ensuing week after infection, 5 older and 4 younger mice succumbed to sepsis or were euthanized, for a total of 19 older and 21 younger mice included in final analysis. The dissemination of the bioluminescent bacteria was noninvasively and longitudinally monitored specifically at the sites of the operative (op) right legs (possessing the surgically placed orthopaedic implant) and the contralateral non-operative (non-op) left legs using whole *in vivo* BLI for 28 days with imaging performed on days 0, 3, 7, 14, 21, and 28 (Fig. 1a, b). In older mice, the *in vivo* BLI signals from the op legs (that peaked on day 3 (8.97 × 10^4^ ± 2.64 × 10^4^), decreased on day 14 and remained relatively stable through day 28) were substantially greater than the non-op legs (which increased on day 3 (5.45 × 10^3^ ± 1.60 × 10^3^), peaked on day 14 (1.03 × 10^4^ ± 3.60 × 10^3^) and remained stable through day 28) for the entire 28-day course of infection (Fig. 1a, b). In younger mice, although there was a trend for increased *in vivo* BLI signals in the op legs (which increased on day 3 (5.12 × 10^4^ ± 2.20 × 10^4^) and continued to increase through day 21) compared with the non-op legs (which increased on day 3 (1.68 × 10^4^ ± 6.62 × 10^3^) and further increased between days 14 and 28), this did not reach statistical significance (*P* = 0.2143) (Fig. 1a, b). Of note, in the younger mice, *in vivo* BLI signals of the op and non-op were virtually identical on day 28. When the *in vivo* BLI data from an early time point (day 3) were graphed in Euler diagrams, in older mice, there was a clear predilection for the presence of *in vivo* BLI signals (above the background limit of detection [LOD] of 3.2 × 10^3^ photons/s/cm^2^/sr) in the op legs versus the non-op legs (Fig. 1c). By contrast, in younger mice, most of the mice had *in vivo* BLI signals from both the op and non-op legs (Fig. 1c). Taken together, these data indicate that the older mice had a predilection for developing HOIAI in the op legs (with higher *in vivo* BLI signals and numbers of mice with the presence of *in vivo* BLI signals) compared with the non-op legs, whereas the predilection for developing HOIAI in the op legs compared with the non-op legs in younger mice was substantially reduced.

### Body weights during the *S. aureus* bacteremic infection

The body weights of the older and younger mice were also measured on the same days when *in vivo* BLI was performed (Fig. 1d). As expected, the older mice had higher starting body weights than the younger mice due to the gain of body weight as they aged (28.48 ± 2.86 g versus 25.35 ± 1.80 g, respectively). However, both groups had an almost identical maximal percent decrease in body weights of 22.89 ± 1.83% for the older mice and 19.29 ± 3.75% for younger mice on day 7 following the intravenous *S. aureus* bacteremic challenge. In addition, both the older and younger mice had increased their body weights as the systemic infection resolved between days 7 to 28, approaching the baseline body weights for both groups of mice. These data indicate that both the older and younger mice developed a similar degree of severity of the bacteremic infection and concomitant resolution of the systemic infection, despite any differences observed in the *in vivo* BLI signals from the op and non-op legs in Fig. 1.

### *Ex vivo* CFU enumeration

**Fig. 2:**
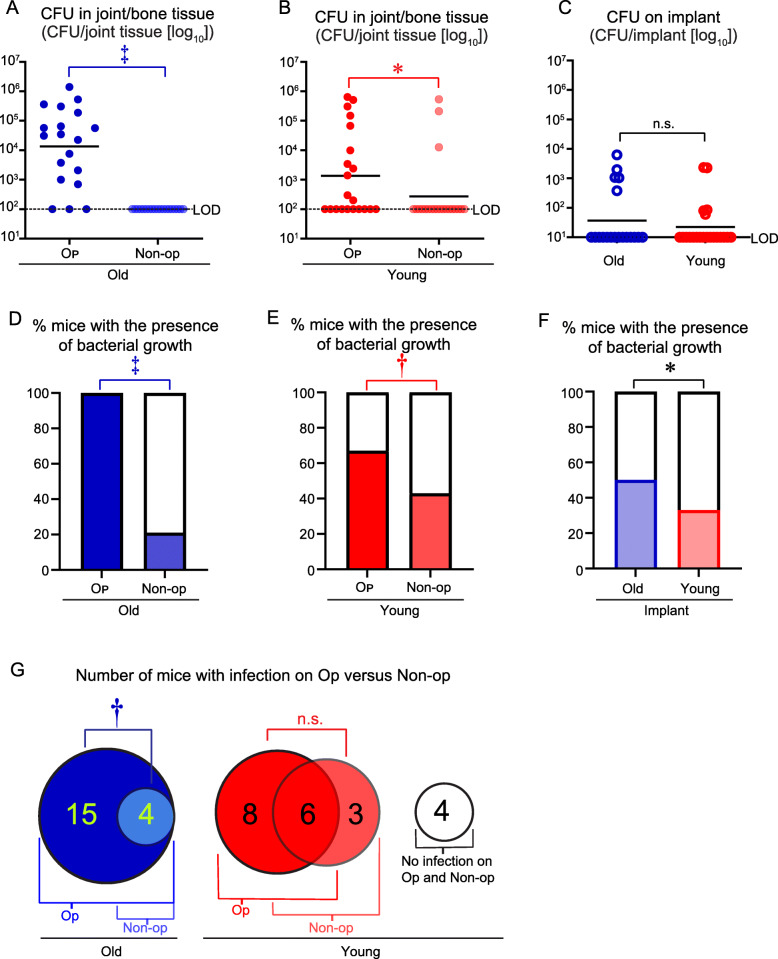
*Ex vivo* CFU harvested from bone/joint tissue specimens and implants from older and younger mice. On day 28 after the intravenous bacterial inoculation, mice were euthanized, and *ex vivo* CFU were isolated from bone/joint homogenates and from sonicated implants from the operative (op) and non-operative (non-op) legs of older and younger mice (*n* = 19–21 mice/group). **a**–**c** *Ex vivo* CFU from older mice (**a**), younger mice (**b**), and implant specimens (**c**). **d**–**f** Percentage of mice with the presence or absence of bacteria growth after 48 h of additional culture of bone/tissue homogenates of older mice (**d**), younger mice (**e**), and implant specimens (**f**). **g** Euler diagram depicting the number of mice on day 28 with and/or without detectable *ex vivo* CFU from the bone/joint tissue, implant specimens and broth cultures. *P < 0.05, †*P* < 0.01, and ‡*P* < 0.001, between op versus non-op bone/joint tissue specimens (**a**, **b**) or older versus younger mice (**c**) as calculated by 2-tailed Mann-Whitney *U* test or between the percentage of specimens with or without bacterial growth, as calculated by 2-tailed Fischer’s exact test (**d**–**g**). *n.s.* not significant

The experiment was arbitrarily ended on day 28 after the intravenous *S. aureus* inoculation, and all mice were euthanized and the bone/joint tissue and implants were harvested to enumerate the *ex vivo* CFU as an additional approach to confirm the findings obtained using *in vivo* BLI (Fig. 1a, b). In older mice, the *ex vivo* CFU isolated from the bone/joint tissue from the op legs (1.62 × 10^5^ ± 7.68 × 10^4^) were more than 100-fold statistically higher than the non-op legs (1.00 × 10^2^, which was the LOD) (*P* < 0.001) (Fig. 2a). In the younger mice, the *ex vivo* CFU isolated from the bone/joint tissue from the op legs (8.08 × 10^4^ ± 3.95 × 10^4^) were only ~ 2-fold statistically higher than the non-op legs (3.65 × 10^4^ ± 2.71 × 10^4^) (*P* < 0.05) (Fig. 2b). Both older and younger mice had similar *ex vivo* CFU isolated from the implants, which did not significantly differ from each other (Fig. 2c). Next, to determine whether there were any bacteria present in the op versus the non-op legs in older and younger mice, the bone/joint tissue and implant specimens were cultured for 48 h in broth culture and the presence/absence of CFU was evaluated. The additional 48-h broth culture has been an established approach to detect *S. aureus* growth, including slow-growing and small-colony variants associated with *S. aureus* biofilms [35]. In older mice, there was a clear predilection as there were many more mice with *ex vivo* CFU present in the bone/joint tissue and/or in the broth cultures from the op (*n* = 19, 100% of the mice) compared with the non-op (*n* = 4, 21% of the mice) legs (Fig. 2d). There were more of the older mice without any detectable *ex vivo* CFU present in the bone/joint tissue of the non-op legs (*n* = 15, 78.9% of the mice). By contrast, in younger mice, most of the mice had *ex vivo* CFU present in the bone/joint tissue when cultured in broth from both the op (*n* = 14, 67% of the mice) and the non-op legs (*n* = 9, 43% of the mice) (Fig. 2e). The older mice also had a slightly increased percentage of mice with *ex vivo* CFU detected from implant cultures compared with the percentage of younger mice (Fig. 2f). When the data for the *ex vivo* CFU and presence/absence of bacterial growth after broth culture for both the bone/joint tissue and/or implant specimens were combined and graphed in Euler diagrams, there again was a clear predilection in older mice for the presence of *ex vivo* CFU in the op (*n* = 19, 100% of the mice) versus the non-op (*n* = 4, 21% of the mice) legs (Fig. 2g). By contrast, in younger mice, most of the mice had *ex vivo* CFU detected in the op (*n* = 14, 66.7% of the mice) and/or non-op legs (*n* = 9, 42.9% of the mice), which were not statistically different from each other. Some of the younger mice (*n* = 4, 19.0% of the mice) also had no infection in either the op or non-op legs. Taken together, these results corroborate with the *in vivo* BLI data (Fig. 1), demonstrating that older mice had a predilection for the development of HOIAI in the op compared with the non-op legs, likely due to the presence of the implant. In contrast, the predilection for the development of HOIAI between the op and non-op legs was substantially less apparent in younger mice.

### X-ray analysis

**Fig. 3:**
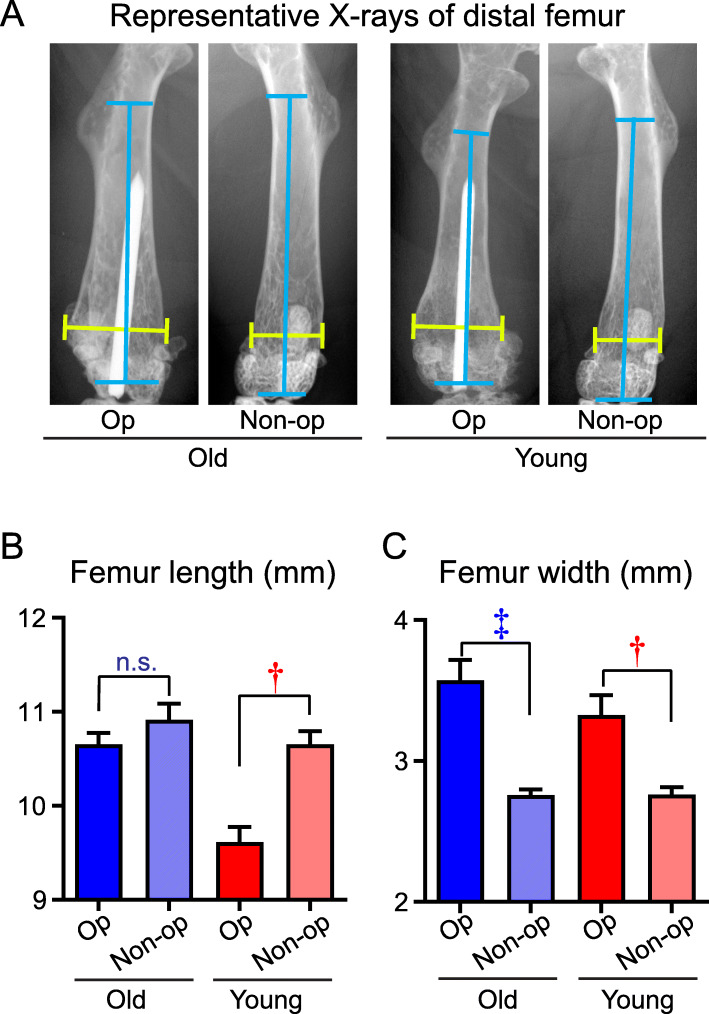
X-ray analysis of femurs of older and younger mice on day 28 after infection. On day 28 after the intravenous bacterial inoculation, mice were euthanized and anteroposterior (AP) X-rays were obtained on the operative (op) and non-operative (non-op) legs of older and younger mice. **a** Representative AP radiographs. Yellow brackets denote femur width and blue brackets denote femur length. **b** Mean femur length measured perpendicular from the line bisecting the 3rd trochanter to the trochlea (mm) ± s.e.m.. **c** Mean femur width measured proximal to the fabella (mm) ± s.e.m.. *P < 0.05, †*P* < 0.01, and ‡*P* < 0.001, between op versus non-op legs in older and younger mice, as calculated by 2-tailed Mann Whitney *U* test (**b**, **c**). *n.s.* not significant

On day 28, anteroposterior (AP) X-rays of the op and non-op legs were obtained to monitor the longitudinal growth, via femoral length of the op versus non-op legs, and also to quantify infection-induced reactive bone changes in both groups via femoral width (Fig. 3a). The markedly increased width of mouse femurs during a *S. aureus* OIAI has been directly correlated with the degree of infection [32–34]. In younger mice, the femoral length was significantly shorter in the op (9.62 ± 0.16 mm) versus non-op (10.65 ± 0.14 mm) legs (*P* < 0.001), whereas in older mice, the femoral length did not significantly differ between op (10.66 ± 0.12 mm) versus non-op (10.92 ± 0.17 mm) legs, and the length was similar to that in the non-op legs of the younger mice (Fig. 3b). In contrast, in both the older and younger mice, the width of the distal femur was statistically greater in the op versus the non-op legs (Fig. 3c). Overall, the decreased length in the op legs of younger mice was likely due to the surgical and/or infectious insult on bone mass and growth velocity of the distal femoral physis in younger mice. In contrast, the older mice had substantially less growth potential in the distal femoral physis, and as such, there was less impact on growth from surgical and/or infectious insult. Meanwhile, the greater infectious burden of the HOIAI that developed in the op legs in both the older and younger mice resulted in increased femoral width compared to the non-op legs, highlighting the relationship between presence of an implant and severity of infection.

## Discussion

Elucidating pathogenesis of HOIAI may aid clinicians in predicting and identifying patients at risk for developing infection, as well as in diagnosing patients with unrecognized established infection. Patterns of infection (hematogenous osteomyelitis versus OIAI) may be influenced by aging as the physis becomes less active with decreased vascularity in adulthood [36–38]. In an established model of preclinical HOIAI [26], we sought to evaluate for any influence of developing infection in older versus younger mice. Our results demonstrate that there was a strong predilection for the development of HOIAI in the operative legs of older mice, whereas in younger mice, the predilection was less significant due to similar rates of infection in both the operative and non-operative legs, thus confirming our hypothesis. These results suggest that in older mice, the susceptibility of developing an infection was largely determined by the presence of an orthopaedic implant, whereas in younger mice, the development of infection was not substantially influenced by the presence of an orthopaedic implant. We theorize that these findings are likely due to the less metabolically active and less vascular physis in older versus younger mice, which will be directly evaluated in our future work.

Although biofilm formation on the implants has been elucidated as an important factor in developing OIAI [39–44], the pathogenesis of HOIAI remains largely unknown. In hematogenous osteomyelitis and perhaps HOIAI, it is thought that bacteria from the bloodstream transit across the endothelium in the bone particularly in the metaphyseal region where there is turbulent flow [19, 20], terminal metaphyseal vessels that are open ended and permit bacterial entry [10, 12, 21], and where gaps in the endothelium are present [10, 12, 21]. Subsequently, the bacteria adhere to various surfaces (implant, bone, and/or tissue) [45]. Orthopaedic implants are particularly susceptible to biofilm formation because these inorganic materials lack the ability to trigger immune-mediated bacterial clearance, and antibiotic penetration is limited since the materials do not have a direct blood supply [1, 41, 42, 46, 47]. Given that pediatric patients, most often without any orthopaedic implants, are more susceptible to septic arthritis and osteomyelitis from a hematogenous source than adult patients [9–12], an active physis even in the absence of an implant is likely a crucial factor in driving the development of a hematogenous musculoskeletal infection. Our results that the hematogenous infection in younger mice occurred more similarly in both the op and non-op legs supports this hypothesis. Although it is unclear why this is the case, younger mice have a more vascularized metaphysis required to supply nutrients for bone growth [14–18] compared with the more skeletally mature metaphyseal region in older mice [22, 23, 48, 49]. In addition, bacteria might persist in this region without causing a robust infection, evidenced by the relatively higher percentage of younger mice with the presence of bacteria in the bone/joint tissue of the non-op legs determined by positive broth cultures (33%), despite varying levels of *in vivo* BLI signals and *ex vivo* CFU in the same non-op legs. Based on the 28-day timeframe of monitoring the infection in this study, it is unclear whether these low levels of lingering bacteria in the bone/joint tissue of the non-op legs could at some later time point lead to acute infection, remain viable but not cause overt infection, or ultimately be cleared by the host immune system. In contrast, in the older mice with a more skeletally mature metaphyseal region, the non-op legs were more resistant to bacterial seeding alone, based on the low number of bone/joint tissue specimens with positive broth cultures (21%). In older mice and potentially human adults, the ability of the bacteria to form biofilms on the implanted foreign materials (in the absence of the increased growth velocity seen in the physis of younger mice) [1, 46] was likely the most important determinant for developing HOIAI. Prior work on HOIAI confirmed that surgery alone without an implant present does not lead to significant musculoskeletal infection derived from a hematogenous source [26]. The presence of an implant may also confer increased bacterial accessibility to bone in older mice, in which regional and/or cellular architecture may be more adversely impacted or have slower healing potential, compared with younger mice. Taken together, this study shows that the two major determinants of hematogenous musculoskeletal infection are the age of the mice (likely related to the metabolic activity and vascularity of the physis) and the presence of an orthopaedic implant.

The temporal dynamics of the infection also appear to be influenced by both the age of the mice and presence of an orthopaedic implant. The younger mice had earlier infiltration of bacteria in non-op legs, as evidenced by a high *in vivo* BLI signal already on day 3 (1.68 × 10^4^ ± 6.62 × 10^3^). In contrast, the non-op legs of older mice had half the amount of *in vivo* BLI signal (5.45 × 10^3^ ± 1.60 × 10^3^), which increased more slowly as the infection progressed. Thus, it could be that the relatively increased metabolic activity and vascularity of the physis in younger mice led to enhanced bacterial dissemination to the non-op legs, compared with older mice with a lesser degree of metabolic activity and vascularity of the physis. Furthermore, peak infection was reached early (by day 3) in the operative legs of both the older and younger mice, while peak infection was not reached in non-operative legs until late (weeks 2–4), suggesting that the presence of an implant can expedite and amplify infection. Thus, HOIAI may be of clinical concern in the first few days after bacteremia, while hematogenous osteomyelitis may more likely manifest a few weeks after bacteremia. Future studies could assess earlier and more frequent BLI time points to further expound these temporal differences.

There are several limitations to this study. First, unlike humans, the physis in mice never fully fuses with age [50], making it impossible to compare mice with an active physis to mice with a fully fused physis. Although long bone growth may continue up to 6 months [51], they nevertheless do not substantially grow in length after 12 weeks of age, which was the minimal age used in the older mice experimental group, as evidenced by the minimal change in femoral lengths between the older operative and non-operative legs. The younger mouse group of 5–8 weeks of age was chosen because the K-wires could not be placed in the small femurs of mice younger than 5 weeks, and the older mouse group of 14–21 weeks of age was chosen because this age was beyond the first 12 weeks of age when the most rapid growth of the long bones has already occurred. In the future, to overcome this limitation, we plan to build on these initial findings by evaluating mice with more disparate ages and using an alternative animal model of HOIAI in which the physis fully fuses. Similarly, although metabolic activity and vascularity were not directly assessed in this study, the radiographs demonstrated a significant difference in growth velocity via length between the op and non-op legs in younger but not older mice. This result likely is due to the differential bone growth activity in the physis between these two age groups as a result of the infectious and inflammatory insult on longitudinal growth, thus functioning as an indirect assessment of metabolic activity. Third, the precise region of bacterial dissemination to the joint, epiphysis and/or metaphysis and whether the primary infection in the op or non-op sites involved septic arthritis or osteomyelitis was not possible with the limited spatial resolution of *in vivo* BLI. Unfortunately, histological analysis is limited to specific time points on euthanized mice. Perhaps more sophisticated imaging technologies such as 2-photon microscopy could be attempted but is beyond the scope of this initial report. Fourth, it is likely that different bacterial virulence mechanisms such as collagen-binding adhesin (Cna), extracellular adherence protein (Eap), lipoteichoic acid (LTA), wall teichoic acid (WTA) and fibronectin binding proteins A and B (FnBPA and FnBPB) [45] contributed to facilitating the transit of the bacteria through the endothelium in this mouse model of HOIAI as many of these bacterial factors have been implicated in the pathogenesis of osteomyelitis and septic arthritis [52–57]. These mechanisms will be the subject of our future work. Finally, pathogenesis of HOIAI could differ between mice and humans, and likelihood of developing musculoskeletal infection may be higher in this study, especially since the high intravenous bacterial inoculum (LD10-15) was used, requiring future studies in humans to translate these findings.

In conclusion, development of HOIAI was influenced by the age of the mice as well as the presence or absence of an orthopaedic implant. Our results warrant further investigation in humans that might guide clinical monitoring after a bacteremic exposure because, based on this study, adult patients may have an elevated risk of developing HOIAI, whereas pediatric patients may be at risk for developing a musculoskeletal infection whether or not an orthopaedic implant is present. Future studies with histologic analysis and other advanced imaging technologies to elucidate the microscopic dissemination of hematogenous bacteria to the bone and joint, assessment of earlier and more frequent time points to further evaluate the temporal differences during HOIAI in younger and older mice, as well as studies in larger animals and humans, will help further elucidate the role of age and other factors in the pathogenesis of HOIAI.

## Data Availability

All data generated or analyzed during this study are included in this published article.

## Abbreviations

OIAI: Orthopaedic implant-associated infection
HOIAI: Hematogenous orthopaedic implant-associated infection
BLI: Bioluminescence imaging
MRSA: Methicillin-resistant *Staphylococcus aureus*
Op: Operative
Non-op: Non-operative
AP: Anteroposterior
